# Evaluating a web-based paediatric infectious diseases journal club: more than just critical appraisal?

**DOI:** 10.1186/s12909-014-0242-9

**Published:** 2014-11-14

**Authors:** Asha C Bowen, Tom G Connell, Penelope A Bryant

**Affiliations:** Department of General Paediatrics, Princess Margaret Hospital, Perth, WA Australia; Child Health Division, Menzies School of Health Research, Charles Darwin University, Darwin, NT Australia; Department of General Medicine, Infectious Diseases Unit, The Royal Children’s Hospital, Melbourne, VIC Australia; Murdoch Children’s Research Institute, Melbourne, VIC Australia

**Keywords:** Journal club, Medical education, Web-based, Paediatrics

## Abstract

para

## Dear editor,

We describe the development and evaluation of a web-based, paediatric infectious diseases (PID) journal club (JC). A coordinated roster with monthly submissions from 11 clinical sites has increased knowledge-sharing, member-engagement and opportunities for trainees to be mentored in critical appraisal writing. The JC is now in its third year with ongoing educational value to this sub-specialty group.

Reported characteristics of successful JC include making attendance mandatory, small programs, teaching critical appraisal skills, having a moderator trained in critical appraisal and making food available [1,2]. We describe an online JC initiative by the Australia and New Zealand Paediatric Infectious Diseases (ANZPID) group that has none of the above attributes.

In December 2011, ANZPID, the paediatric special interest group of the Australian Society of Infectious Diseases (ASID), started a JC page on the ASID website. After 6 months, an online JC was formally established (www.asid.net.au/groups/paediatric-id-journal-club) with a PID specialist as the coordinator. JC appraisals are submitted by ANZPID members and it is primarily aimed at this group, but the website is available to anyone at the above URL. This international collaboration of a subspecialty group is a novel method for sharing knowledge and engaging members of which there are no examples in the paediatric literature and none with an evaluation. To assess the value and impact of this online JC after 12 months of activity, we analysed traffic through the JC webpages, and surveyed participants via the ANZPID email list in June 2013, which includes 115 addresses of whom 40 are active members. Active membership was defined as PID consultants, trainees and others on the mailing list who are involved in any ANZPID activities (e.g. meetings, forum discussions).

Through a co-ordinated roster, each month a different hospital in Australia or New Zealand with a PID specialist critically appraises a recently-published article. The only pre-requisites are that the article must be peer-reviewed in an online or paper journal and that it is relevant to the practice of PID physicians. The 11 clinical sites each have at least one PID physician and trainees. These sites include stand-alone children’s hospitals and general hospitals admitting children and geographically cover the continent of Australasia. At the start of each month, the coordinator sends a weekly email to remind the submitting site of their opportunity to contribute. Submissions are always timely and in two years of operation, there has not been a missed submission. Indeed, some sites have recently submitted two or three appraisals. The appraisal links to the article and summarises the background, main findings and clinical practice implications. The first 30 JC items have appraised: case–control or cohort studies (16, 53%), randomised controlled trials (7, 23%), case series (4, 13%) and systematic or literature reviews (3, 10%). Commentaries were occasionally included alongside the article(s) they were commenting upon. The appraisal is uploaded each month to the JC section of the ASID website, where it remains permanently [3]. ANZPID members receive an email alert of new items.

**Figure 1:**
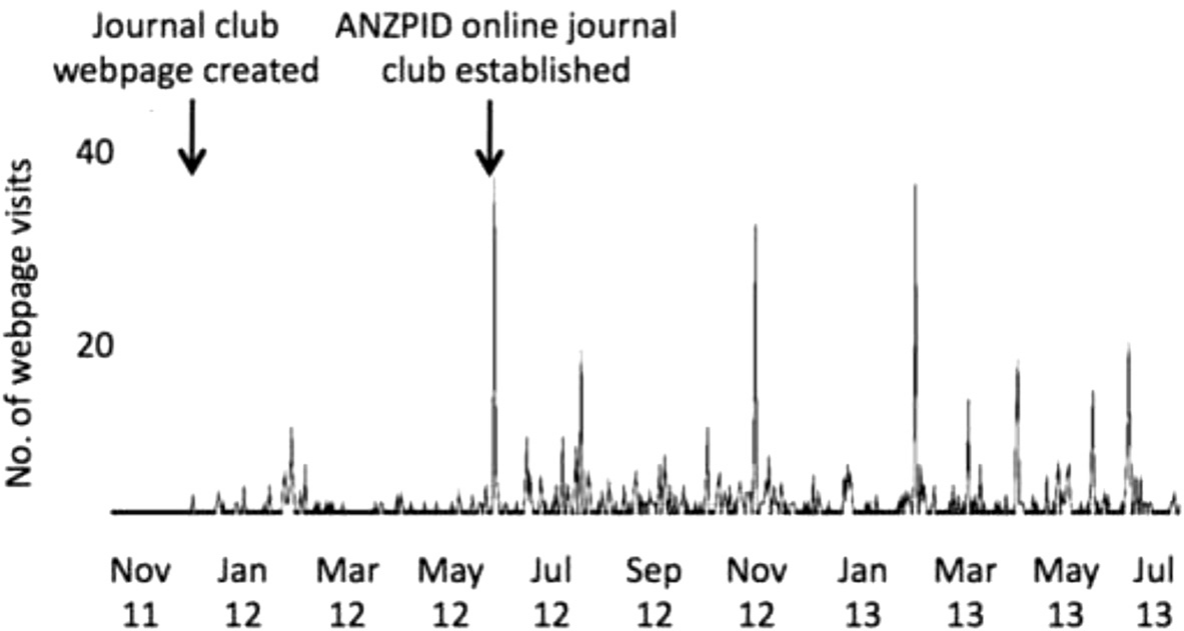
**Number of journal club webpage visits to the ANZPID website from November 2011 to July 2013.**

In the initial 6-month period after the JC webpage creation, three JC items were uploaded and there were 50 visits to the webpage (8 hits/month). In the 12 months following the online JC launch, 11 items were uploaded and there were 548 visits to the webpage (46 hits/month), an almost 6-fold increase. The average time spent on the JC webpage was 1 min 58 sec, almost 30% longer than the average 1 min 31 sec per page for the whole website. The greatest number of hits occurred after the email alert (Figure 1). Consultants submitted 75% of items and trainees 25%. It is an excellent opportunity on both sides for experts in critical appraisal to mentor trainees to select a topical article and write a concise submission.

There were 25/40 (63%) survey responses. For 18/25 (72%), this was the first time they had participated in an online JC as contributors (7/25, 28%) or to read content (11/25, 44%). In the first 12 months, over 50% (13/25) respondents had read more than three JC submissions. Respondents reported that they used submissions for guiding clinical management (60%), teaching (28%), academic discussions (56%), subsequent JC (24%) or research (4%). 75% felt the online JC was an endeavour worth pursuing.

In a recent systematic review on JC effectiveness, [2] no paper reported on the potential translation of evidence from JC into clinical practice, and for the first time we show 60% of responding participants reporting their intention of using the evidence in this way. By having regular submissions appraising relevant articles, sharing the workload amongst a motivated group and using the internet for dissemination, we have shown that lack of a trained moderator need not be an impediment to a successful JC. In addition to continuing professional development, by attracting participants to the subspecialty group website, it provides networking opportunities across a huge geographical area, a potentially valuable resource for other subspecialty groups. We plan to add a forum for discussion of the JC submissions, moderated by the JC co-ordinator. The approximately two minutes it takes busy clinicians to read the short appraisals seems to be time well spent. We have not yet worked out how to make food available.
